# Synthesis, Characterization, and Enzyme Conjugation of Polycaprolactone Nanofibers for Tissue Engineering

**DOI:** 10.3390/pharmaceutics17080953

**Published:** 2025-07-23

**Authors:** Chandana B. Shivakumar, Nithya Rani Raju, Pruthvi G. Ramu, Prashant M. Vishwanath, Ekaterina Silina, Victor Stupin, Raghu Ram Achar

**Affiliations:** 1 Division of Biochemistry, School of Life Sciences, Mysuru, JSS Academy of Higher Education and Research, Mysuru 570015, Karnataka, India; chandanabs@jssuni.edu.in (C.B.S.); nithyaranir@jssuni.edu.in (N.R.R.); pruthvigr@jssuni.edu.in (P.G.R.); 2 Department of Biochemistry, JSS Medical College, JSS Academy of Higher Education and Research, Mysuru 570015, Karnataka, India; prashantv@jssuni.edu.in; 3 I.M. Sechenov First Moscow State Medical University (Sechenov University), 119991 Moscow, Russia; silinaekaterina@mail.ru; 4 Department of Hospital Surgery, Pirogov Russian National Research Medical University, 117997 Moscow, Russia; stvictor@bk.ru; 5 Department of Biochemistry, Mahayogi Gorakhnath University Gorakhpur, Gorakhpur 273007, Uttar Pradesh, India

**Keywords:** nanofibers, scanning electron microscope, polycaprolactone, enzymatic, FTIR, electrospinning, wound healing

## Abstract

**Background/Objectives**: A nanostructured membrane of polycaprolactone (a synthetic polymer) was synthesized using an electrospinning technique aiming to enhance its hydrophilicity and rate of degradation by surface modification via aminolysis. Since polycaprolactone nanofibrous films are naturally hydrophobic and with slow degradation, which restricts their use in biological systems, amino groups were added to the fiber surface using the aminolysis technique, greatly increasing the wettability of the membranes. **Methods**: Polycaprolactone nanofibrous membranes were synthesized via the electrospinning technique and surface modification by aminolysis. Trypsin, pepsin, and pancreatin were conjugated onto the aminolyzed PNF surface to further strengthen biocompatibility by enhancing the hydrophilicity, porosity, and biodegradation rate. SEM, FTIR, EDX, and liquid displacement method were performed to investigate proteolytic efficiency and morphological and physical characteristics such as hydrophilicity, porosity, and degradation rates. **Results**: Enzyme activity tests, which showed a zone of clearance, validated the successful enzyme conjugation and stability over a wide range of pH and temperatures. Scanning electron microscopy (SEM) confirms the smooth morphology of nanofibers with diameters ranging from 150 to 950 nm. Fourier transform infrared spectroscopy (FTIR) revealed the presence of O–H, C–O, C=O, C–N, C–H, and O–H functional groups. Energy-dispersive X-ray (EDX) elemental analysis indicates the presence of carbon, oxygen, and nitrogen atoms owing to the presence of peptide and amide bonds. The liquid displacement technique and contact angle proved that Pepsin-PNFs possess notably increased porosity (88.50% ± 0.31%) and hydrophilicity (57.6° ± 2.3 (L), 57.9° ± 2.5 (R)), respectively. Pancreatin-PNFs demonstrated enhanced enzyme activity and degradation rate on day 28 (34.61%). **Conclusions**: These enzyme-conjugated PNFs thus show improvements in physicochemical properties, making them ideal candidates for various biomedical applications. Future studies must aim for optimization of enzyme conjugation and in vitro and in vivo performance to investigate the versatility of these scaffolds.

## 1. Introduction

Every year, many individuals experience one or another type of tissue damage or tissue loss, either due to trauma or illness. The human body possesses a limited regenerative capability, so there is a high demand for tissue-regenerative replacements. In recent years, scientists have been highly motivated to design safe and reliable sources of tissue substitutes [1,2]. Many techniques and improved therapies that regenerate tissue have emerged, and numerous alternatives are under study. Skin damage presents significant treatment challenges, and clinical obstacles such as ongoing bacterial infection and inflammation are present in the care of skin wounds. It is, therefore, essential to produce dressings that are detachable and have inherent multifunctional features [3,4].

To create biological replacements that preserve, repair, or enhance tissue function, the interdisciplinary field of tissue engineering integrates concepts from biology, engineering, and materials science. The goal of tissue engineering is to build functional systems that can replace or heal damaged tissues and organs by combining cells, scaffolds, and bioactive chemicals. This strategy presents viable substitutes for conventional reconstructive methods, possibly overcoming restrictions related to the availability of donor tissue and immunological rejection [5,6].

Tissue engineering has the potential to transform regenerative medicine by tackling important issues in healthcare, like the shortage of donor organs and the limitations of prosthetic devices, by offering remedies for tissue and organ failure. It is utilized in several medical specialties, such as wound healing, cardiovascular medicine, and orthopedics, to provide novel treatments for diseases which currently have no reliable treatments [7].

Three essential elements are involved in tissue engineering: cells, scaffolds, and bioactive substances. (1) Cells: The use of stem cells and differentiated cells is crucial to tissue engineering because they can differentiate into diverse cell types and, thus, are beneficial for creating specific tissues. Recent advances have focused on optimizing cell sources and improving methods for cell proliferation and differentiation in order to improve tissue regeneration [5]. (2) Scaffolds: Scaffolds facilitate cell adhesion, proliferation, and differentiation by providing a three-dimensional structure. Biomaterial advancements have yielded scaffolds with enhanced mechanical and biocompatibility characteristics. By creating intricate scaffold designs that closely resemble the natural extracellular matrix, technologies such as 3D printing and nanotechnology have improved tissue integration and function [8]. (3) Bioactive substances: Bioactive compounds are essential for controlling cell activity and fostering tissue growth. It has been demonstrated that adding growth factors to tissue-engineered constructs improves tissue development by promoting cell proliferation and differentiation. In order to maximize the release of these components and enhance therapeutic results, currently, researchers are investigating controlled delivery systems [9].

In recent years, polymers have gained increased importance in designing tissue scaffolds, although they have major limitations, such as poor mechanical strength, potential immune responses, hydrophobicity, and degradation issues [10]. Polycaprolactone (PCL) is a semi-crystalline biodegradable polyester with a low melting point of 60 °C, produced by ring-opening polymerization of ε-caprolactone. It has good biocompatibility and permeability [11,12,13]. The drawbacks of PCL, such as hydrophobic nature and long degradation time, can be overcome by combining it with other polymeric materials; such polymers have been widely used in the field of biomedical engineering. The biomedical applications of PCL include sutures, drug delivery agents, wound healing, implants, skin tissue engineering, bone engineering, and the development of various tissue grafts [14,15].

PCL has promising applications in bone regeneration predominantly due to its mechanical strength and minimal degradation rate. Effective treatments for bone injuries have been made possible by 3D-printed PCL composites, which provide individualized structures that promote osteogenesis and mimic the extracellular matrix [16,17]. Electrospun PCL-based materials have been employed in skin tissue regeneration because they can replicate the fibrous structure of the natural extracellular matrix [18]. These scaffolds enhance cell adhesion and proliferation, promoting efficient wound healing, and are also used in craniofacial tissue engineering for surgeries both inside and outside the mouth. Their compatibility with the body and their structure make them optimal for repairing complex facial bones [19].

*Burkholderia cepacia* lipase-loaded PCL scaffolds have shown promise in promoting scarless skin tissue regeneration. In vivo studies demonstrate that enzyme-loaded scaffolds effectively promote angiogenesis, reduce inflammatory responses, and mitigate scarring, thereby enhancing the healing process of chronic wounds [20]. However, despite these noteworthy studies, the regenerative potential of common proteolytic enzymes, such as pepsin, trypsin, and pancreatin, remains unexplored pertaining to scaffold-based therapeutic applications. Based on this gap, the current study explores a novel strategy to construct PCL scaffolds integrated with enzymes. Given the most crucial part of proteolytic enzymes in the wound microenvironment, this study aims to investigate their physical and chemical characteristics for application in various wound healing and tissue regeneration contexts.

## 2. Materials and Methods

### 2.1. Synthesis of Polycaprolactone Nanofibers Films

The 10% (*w*/*v*) polycaprolactone (Sigma-Aldrich, St. Louis, MO, USA) with an average molecular weight (Mn) of 80,000 was dissolved in a mixture of solvents using chloroform and methanol in a ratio of 8:2. The solution was stirred on a magnetic stirrer for about 5–6 h. The solution was then loaded into a 5 mL syringe, and the PCL nanofibers were synthesized at the optimized electrospinning parameters, such as a flow rate of 0.18 mL/min, a voltage of 15 kV, and a distance of 8 cm from the tip of the needle to the collector at room temperature, 28 °C. The process was conducted at an ambient RH of 45–50%. Thus, obtained nanofibrous films were stored at 4 °C for further analysis [21].

### 2.2. Conjugation of Enzymes to Polycaprolactone Nanofiber Films

The synthesized PCL nanofibrous films (PNF) were soaked for approximately 10 h at 37 °C in 10% 1,6-hexanediamine solution prepared using isopropanol. Thereafter, the films were gently washed in distilled water 2–3 times to remove the excess amine from the surface of the films and then dried at 37 °C. Meanwhile, three different enzyme solutions containing 5 mg/mL concentration of trypsin, pepsin, and pancreatin were prepared using 0.1 M phosphate buffer of pH 7.4. Three dried PNFs were then soaked in the respective enzyme solutions and incubated at 37 °C for about 10 h to initiate enzyme conjugation onto the films. Later, the nanofibrous films conjugated with trypsin (Try-PNF), pepsin (Pep-PNF), and pancreatin (Pan-PNF) were removed, washed with distilled water, dried (37 °C), and stored at 4 °C until further investigation [22,23].

### 2.3. Confirmation of PCL–Enzyme Conjugation

To confirm conjugation of enzymes onto nanofibers, Coomassie Brilliant Blue (CBB R-250) staining method was followed as described by Zhang et al. (2021) [24], using casein media (1% *w*/*v* casein; 1.8% agar). Simultaneously, qualitative analysis of the proteolytic efficiency of enzyme-conjugated PNFs was performed as described by Robinson et al. (2020) [25], with minor modifications, using 2% agar solution containing skim milk powder (5%). Both casein and skim milk powder mixtures were poured onto different Petri plates and allowed to solidify at room temperature. After the media solidification, wells of 5 mm were made with sterile cork borer. The PNFs were cut into small pieces of 5 mm diameter discs using sterile scissors and placed in each well according to the prior markings. Later, plates were incubated at 37 °C for 24–48 h and observed for the zone of clearance (mm in diameter). The digitization of the clear zones of casein-agar plates was analyzed using ImageJ v1.51j8 and OriginPro 2025 SR1 software [25].

### 2.4. Physical Characterization of Enzyme-Conjugated PCL Nanofibers

#### 2.4.1. Scanning Electron Microscopy

Scanning electron microscopy (SEM; EVO LS 10, ZEISS, Oberkochen, Germany) was performed to examine the morphological and structural characteristics such as pores, bead formation, or nanofiber structures of PNFs (Try-PNF, Pep-PNF, and Pan-PNF). Before observation, PNFs were mounted onto metallic stubs using carbon adhesive tape covered with a layer of gold under a vacuum. The images obtained were analyzed using ImageJ software to calculate the diameter of the fibers and assess morphology [26].

#### 2.4.2. Fourier Transform Infrared (FTIR) Spectroscopy

Fourier transform infrared (FTIR) spectroscopy was used to analyze the functional group composition of PNFs. FTIR spectra of the PNFs (Try-PNF, Pep-PNF, and Pan-PNF) were obtained (PerkinElmer, Shelton, CT, USA) in the range of 4000–400 cm^−1^ in transmission mode. The FTIR spectroscopy data generation and analysis are conducted using PerkinElmer Spectrum IR, version 10.7.2 software.

#### 2.4.3. Energy-Dispersive X-Ray

The energy-dispersive X-ray spectroscopy (EDX) technique was employed to analyze the elemental composition of PNFs. The enzyme-conjugated films (Try-PNF, Pep-PNF, and Pan-PNF) were coated with gold to enhance conductivity and examined using a field emission scanning electron microscope (FE-SEM). The samples were captured at different magnifications at an acceleration voltage of 20 kV. Elemental analysis of the different electrospun membranes was performed using the EDX attachment (EDAX-AMETEK Materials Analysis Division, Mahwah, NJ, USA).

#### 2.4.4. Contact Angles

A Kyowa contact angle meter (Japan) was used to measure the hydrophilicity or hydrophobicity of the PNFs. A 2 cm^2^ PNF is mounted on the stage. Approximately 2 microliters of liquid (distilled water) were dispensed onto the film and allowed to stabilize on the membrane surface for 10 s prior to measurement to ensure consistent wetting behavior, thus supporting the formation of a contact angle with the PNF at room temperature 23–25 °C. A high-speed camera and FAMAS software version 2.0 were employed to measure the contact angle.

#### 2.4.5. Porosity of PNFs

The open porosity of enzyme-conjugated PNFs was determined using the liquid displacement method described by Heris et al. [27] in triplicate. Ethyl alcohol (EtOH) was preferred as a displacement liquid as it can interpenetrate through the porous PNFs without bulging or shriveling the films. The EtOH-filled container was weighed (*W_e_*_1_) before immersing the weighed PNFs (*W_f_*). After removing the excess EtOH, containers with immersed PNFs were weighed (*W_ef_*) and left undisturbed for an hour. Finally, the PNFs were removed, and the weight of the container with the remaining EtOH was recorded (*W_e_*_2_). This study was conducted in triplicates at room temperature (28 °C). The percentage of porosity of PNFs was estimated using the following equation [28].

$$Porosity\;(\%)=\left(\frac{W_{ef}-W_{e2}-W_{f}}{W_{e1}-W_{e2}}\right)\times 100$$

#### 2.4.6. Rate of Degradation of PNFs

The degradation rate of modified PNFs was assessed using a 0.1 M phosphate buffer of pH 7.4. Each type of PNF was cut into triplicates of equal lengths, weighed, immersed in the phosphate buffer of pH 7.4, and incubated at 37 °C for 30 days. The scaffolds were taken out at selected time intervals (7, 14, 21, and 30 days) and allowed to dry at 40 °C. Their dry weights were recorded and immersed in a freshly prepared buffer solution. The process was repeated at each time interval until day 30. The rate of degradation of PNFs was calculated using the following equation [29].

$$Rate\;of\;degradation\left(\%\right)=\left(\frac{W_{i}-W_{d}}{W_{d}}\right)\times 100$$

*W_i_* = initial dry weight of PNFs;

*W_d_* = weight of PNFs (at each interval).

### 2.5. Mechanical Characterization

The mechanical properties such as tensile strength, elongation break, and modulus were evaluated using a Shimadzu universal texture analyser (EZ-SX) device (Shimadzu Corporation, Kyoto, Japan). PNF scaffolds were cut into approximately 100 mm length and 20 mm width (*n* = 3). Scaffolds were clamped at both ends and subjected to a constant stretching rate of 10 mm/min until they reached the fracture point [30].

### 2.6. Biochemical Characterization of Enzyme-Conjugated PCL Nanofibers

#### 2.6.1. Enzyme Activity of Conjugated PNF

The catalytic efficiency of enzyme-conjugated PNFs was investigated using casein as a substrate. The reaction mixture consisted of 0.5 mL of 0.2 M Tris–HCl buffer (pH 8.5), 0.5 mL of 2% casein, and 1 cm^2^ of the enzyme-conjugated PNFs (Try-PNF, Pep-PNF, and Pan-PNF). The mixture was incubated at 37 °C for 2.5 h. A total of 1 mL of 0.44 M trichloroacetic acid (TCA) was added to the mixture after incubation and allowed to stand for about 30 min at room temperature and centrifuged at 1500 rpm for 15 min at 4 °C. The supernatant was collected, and 2.5 mL of 0.4 M sodium carbonate and 0.5 mL of Folin–Ciocalteu’s reagent were added; then, the mixture was incubated at room temperature for 30 min. The absorbance was taken at 660 nm and results were expressed as “U”. The study was conducted in triplicates and evaluated against tyrosine standard curve. 1U enzyme activity is defined as the amount of enzyme required to produce tyrosine (1 µmol/min) under the given assay conditions [31].

#### 2.6.2. Effect of pH on the Activity of Enzyme-Conjugated PNFs

PNFs were evaluated for their catalytic efficiency at varying H+ ion concentrations using different buffers of varying pH ranges (pH 5.0, 6.0, 7.0, 8.0, and 9.0). The PNFs (Try-PNF, Pep-PNF, and Pan-PNF) were incubated with 0.5 mL buffers and 0.5 mL casein for 2.5 h at 37 °C. As described earlier in enzyme activity, further study was conducted.

#### 2.6.3. Effect of Temperature on the Activity of Enzyme-Conjugated PNFs

The effect of different temperatures on the catalytic activity of PNFs was assessed using the enzymatic activity assay with minor modifications. The reaction mixtures containing PNFs (Try-PNF, Pep-PNF, and Pan-PNF), 0.5 mL of casein, and 0.5 mL of Tris HCl buffer in triplicates were incubated at 4 °C, 25 °C, 37 °C, 50 °C, and 80 °C for 2.5 h. Further studies were conducted as described earlier.

### 2.7. Statistical Analysis

All experiments were conducted in triplicates, a one-way ANOVA Tukey’s comparison test was performed, and results were represented as mean ± SD. A *p* < 0.05 was regarded as statistically significant. The data were analyzed using OriginPro SR1 software.

## 3. Results

### 3.1. Confirmation of Enzyme Conjugation on PNFs

**Figure 1:**
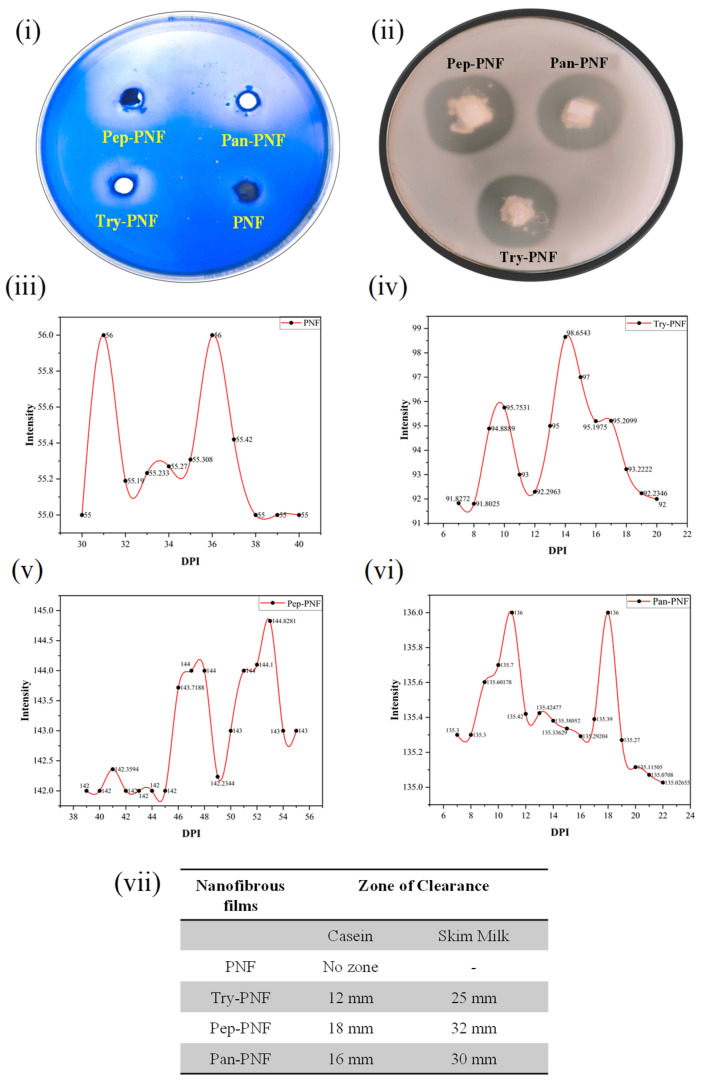
Casein degradation by PNFs: (**i**) casein-agar plate assay. (**ii**) Skim-milk agar plate assay showing wells treated with different PNFs (trypsin-conjugated PNF, pepsin-conjugated PNF, and pancreatin-conjugated PNF (Try-PNF < Pan-PNF < Pep-PNF)). No degradation was observed in the plain PNF disc. (**iii**–**vi**) Intensity profile of zones around the wells of casein-agar plate assay. (**vii**) Nanofiber films and their corresponding zone of inhibition in casein agar and skim milk agar plates.

The caseinolytic plate assay confirms the successful conjugation of enzymes (trypsin, pepsin, and pancreatin) onto the nanofibers without loss of enzyme activity. As shown in Figure 1i,ii, the zone of degradation around wells Try-PNF, Pep-PNF, and Pan-PNF demonstrates the casein degradation, while PNF alone had no zone of clearance due to lack of caseinolytic potential. Analyzed areas around the well using ImageJ and Origin Pro 2025 SR1 software represent that the intensity of the degradation zone correlates with enzyme activity; lighter zones indicate higher degradation due to more active enzymes. The absence of a zone of degradation corresponds to a denser area around PNF’s lack of proteolytic potential due to the absence of enzyme conjugates (intensity peak at 56, 0 mm zone); Figure 1iii. In contrast, Try-PNF in Figure 1iv (intensity peak at 98, 12/25 mm zone) showed moderate degradation, Pan-PNF (intensity peak at 136, 16/30 mm zone) in Figure 1vi showed substantial degradation of casein, and Pep-PNF in Figure 1v (144 intensity, 18/32 mm zone) had the highest casein degradation potential. These findings suggest that pancreatin, trypsin, and pepsin PNFs show significant degradation, confirming the successful conjugation of respective enzymes.

### 3.2. Physical Characterization of Enzyme-Conjugated PCL Nanofibers

#### 3.2.1. SEM Analysis

**Figure 2:**
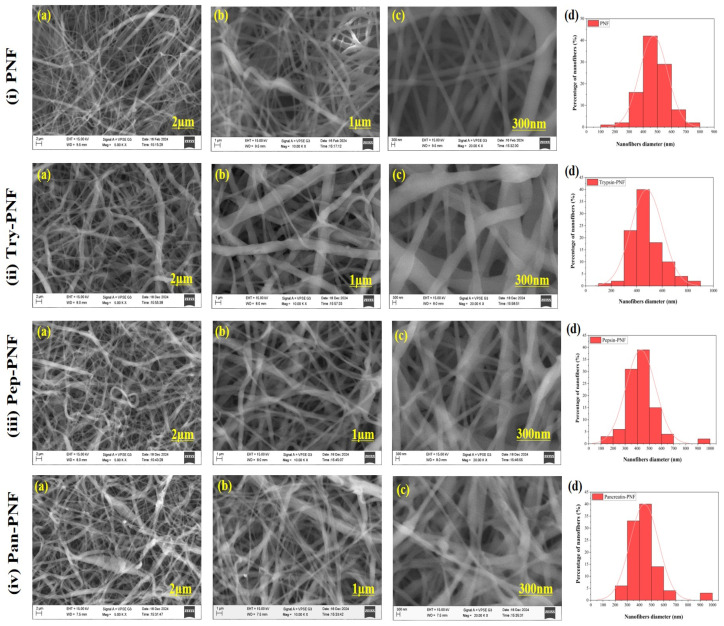
SEM images: (**i**) plain PNF nanofibers (PNF); (**ii**) trypsin-conjugated nanofibers (Try-PNF); (**iii**) pepsin-conjugated nanofibers (Pep-PNF); (**iv**) pancreatin-conjugated nanofibers (Pan-PNF). (**a**–**c**) SEM images at magnifications of 2 µm, 1 µm, 300 nm, respectively, and (**d**) histogram showing the diameter distribution of nanofibers.

The SEM analysis was conducted to determine the morphology and quality of the nanofibers. The SEM images (Figure 2) were captured at different areas of each sample to observe their morphology; eight random photographs from different areas of each sample were captured. The diameter of the nanofibers was measured using ImageJ software. The diameter of PNF, Try-PNF, Pep-PNF, and Pan-PNF was found to be in the range of 150–750 nm, 150–850 nm, 150–900 nm, and 250–950 nm, respectively. The conjugation of enzymes (trypsin, pepsin, and pancreatin) resulted in an increase in the diameter of the nanofibers. Pan-PNF showed the highest diameter among all the others due to their larger molecular structure, followed by Pep-PNF. Regardless of their conjugation and nanofiber width, all the nanofibers possessed smooth morphology and no structural abnormalities.

#### 3.2.2. FTIR Spectra for Functional Group Analysis

**Figure 3:**
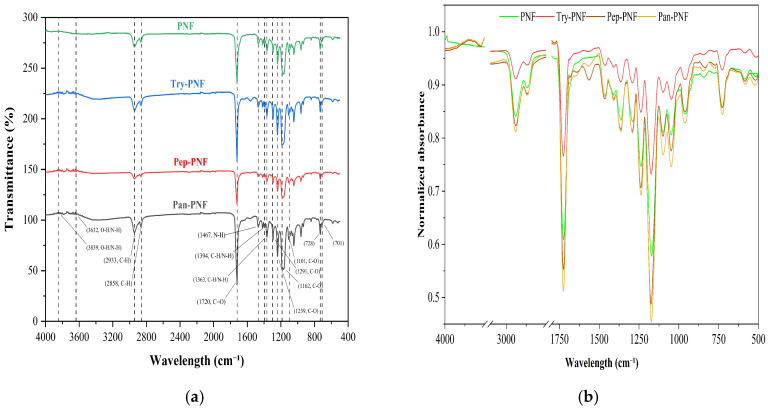
(**a**) FTIR spectrum with functional group analysis. (**b**) FTIR spectral normalization (0–1 scale) by maximum absorbance using OriginPro SR1.

The FTIR spectral analysis was conducted to study the functional groups present in the nanofibers using OriginPro SR1 software. FTIR raw data was primarily baseline corrected and spectral normalization (Scale 0–1) was performed with maximum absorbance. As presented in Figure 3a, in plain PCL nanofiber (PNF), the 2860–2950 cm^−1^ peak corresponds to the C–H stretching vibrations in the aliphatic chains of PCL. PCL is a polymer composed of repeating –CH_2_– and –CH_3_ units. The strong peak around 1725 cm^−1^ is attributed to the stretching vibration of the carbonyl (C=O) group in the ester linkages, a key component of PCL. The 1100–1300 cm^−1^ peak range corresponds to the C–O stretching vibrations of the ester group (–COO–) in the polymer backbone typically occurring in this region. In Try-PNF, Pep-PNF, and Pan-PNF IR spectra, peaks at 3636.87 to 3844.63 cm^−1^ suggest the presence of hydroxyl (O–H) or amine (N–H) functional groups (Figure 3). The multiple peaks could result from hydrogen bonding or different amine environments, confirming aminolysis. The peak at 1720 cm^−1^ indicates a carbonyl group, which could be attributed to ester or amide linkages after aminolysis or peptide bonds in the pancreatin conjugate. The peak ranges of 1559.47 cm^−1^ and 1470.77 cm^−1^ are characteristic of secondary amide groups, confirming peptide or amide bond formation. Peaks at 1240.91, 1186.92, and 1046.38 cm^−1^ correspond to C–O bonds, likely from ester linkages or peptide bonds modified during aminolysis. Peaks at 732.03 and 710.01 cm^−1^ suggest aromatic or cyclic structures, possibly part of the enzyme structure or conjugated residues. Peaks 1366.16 cm^−1^ and 1397.01 cm^−1^ correspond to various bending or stretching modes of C–H or N–H bonds and confirm complex molecular environments in the conjugated system.

#### 3.2.3. EDX Analysis

**Figure 4:**
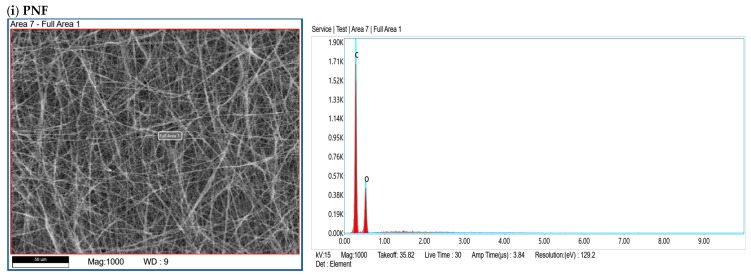

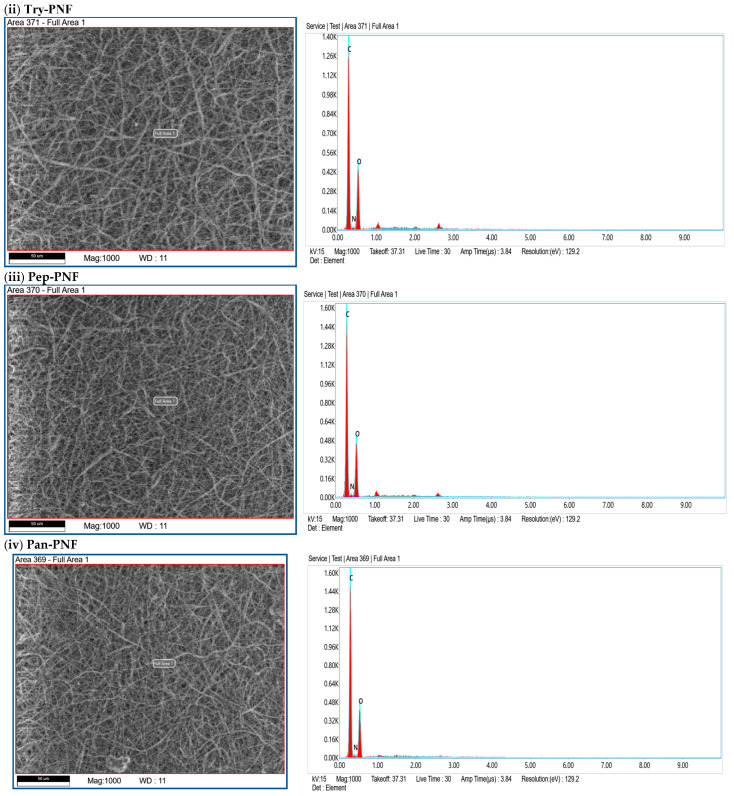
EDX elemental analysis of PNFs showing the confirmation of the enzyme’s conjugation by the presence of nitrogen and increased level of oxygen.

The EDX analysis was performed to determine the elemental composition of PNFs. PNF spectral data showed carbon content of 66.8% and oxygen content of 33.2%, as expected since polycaprolactone is a synthetic polymer composed primarily of carbon-based aliphatic chains and ester groups (–C=O and –C–O–). Nitrogen and other elements are absent, confirming pure PNF nanofibers. The EDX data of Try-PNF (Figure 4 and Table 1) confirm that the polycaprolactone nanofibers have undergone successful aminolysis and trypsin conjugation. The presence of nitrogen (4.4%) and increased oxygen content (36.7%) provides clear evidence of surface functionalization and enzyme conjugation. In Pan-PNF, carbon (59.5%) remains the dominant element. Oxygen (35.5%) emerges from ester groups in PNF, while nitrogen (5%) content confirms the successful introduction of amino groups through aminolysis, providing reactive sites for pancreatin conjugation. The Pep-PNF EDX analysis, like Try-PNF and Pan-PNF, confirms that aminolysis has been effective in modifying the polycaprolactone nanofibers. It also indicates successful conjugation of pepsin, as evidenced by the presence of nitrogen (5.1%) and elevated oxygen (33.8%) content and reduced carbon content (61.2%) compared to unmodified PNF.

#### 3.2.4. Contact Angle Analysis

**Figure 5:**
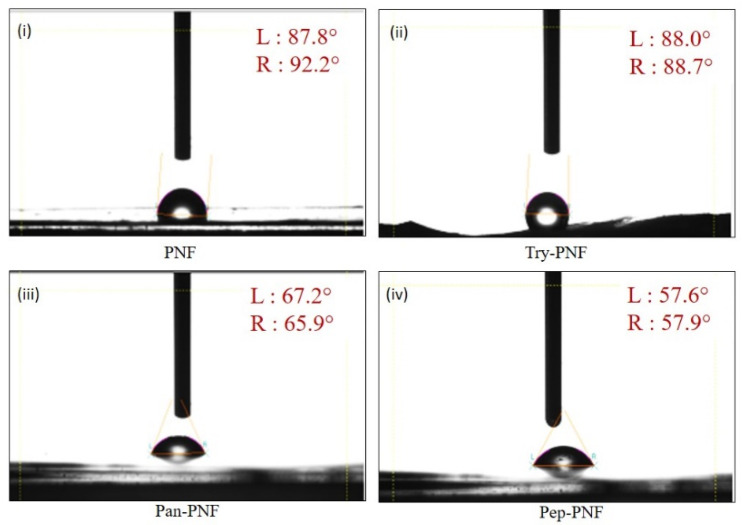
Measurements of contact angle of (**i**) PNF, (**ii**) Try-PNF, (**iii**) Pan-PNF, and (**iv**) Pep-PNF depicting the hydrophilic nature of nanofibrous films where PNF < Try-PNF < Pan-PNF < Pep-PNF.

The surface hydrophilic and hydrophobic properties of PNFs were evaluated using contact angle measurements. Materials with higher contact angles represent more hydrophobic surfaces, while lower contact angles indicate higher hydrophilic surfaces. PNF exhibited a higher contact angle [87.8° (L) and 92.2° (R)], indicating more hydrophobic surfaces. This property limits the application of PNFs in wound healing or tissue engineering where water retention and cell adhesion are critical. Try-PNF [88° (L):88.7° (R)] showed no significant change compared to PNF, while Pan-PNF [67.2° (L):65.9° (R)] exhibited moderate reduction. As represented in Figure 5 and Table 2, Pep-PNF was hydrophilic in nature compared to other PNF conjugates with a contact angle of 57.6° (L) and 57.9° (R).

#### 3.2.5. Percentage Porosity of Nanofibers

**Figure 6:**
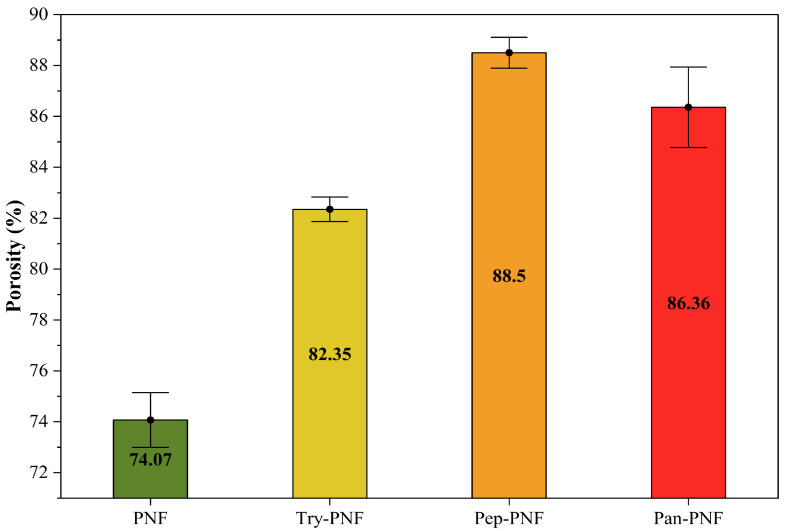
Percentage of porosity in nanofibrous films.

In wound care, dressings are typically designed to be porous to absorb wound exudates and wastes. These porous dressings are also employed to load targeted drugs for treatment. Therefore, the porosity of fabricated PNFs was evaluated using the liquid displacement method. As shown in Figure 6, the porosity of PNF was found to be 74.07% ± 0.55, making it more compact compared to other enzyme-conjugated PNFs. The compact structures of PNFs may limit their application in drug delivery and wound exudate absorption. Try-PNF, Pan-PNF, and Pep-PNF showed increased porosities of 82.35 ± 0.25% (*p* < 0.05), 86.36 ± 0.81% (*p* < 0.05), and 88.50 ± 0.31% (*p* < 0.05), respectively. The increased porosity of these PNFs may be due to enzyme conjugation onto nanofibers, resulting in increased surface area. These results suggest that they may be potential candidates for various applications requiring high fluid absorption, cellular interaction, and tissue engineering.

#### 3.2.6. Rate of Degradation of Nanofibers

**Figure 7:**
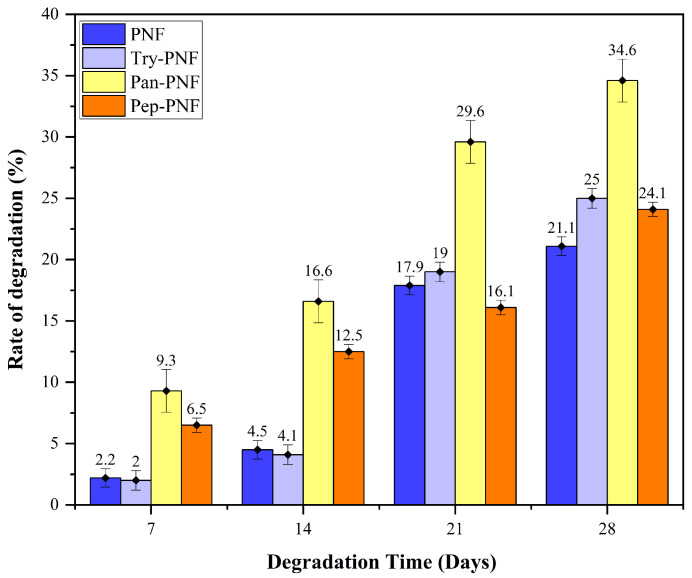
Percentage of degradation of nanofibers, the Pan-PNF showing the highest degradation.

In numerous biomedical applications, biodegradation is one of the key aspects of concern when employing synthetic or natural compounds and materials. In this context, enzyme-conjugated PNFs were assessed for their degradation rate in a controlled environmental (28 days; 37 °C; pH 7). As shown in Figure 7, PNF, Try-PNF, and Pep-PNF rates of degradation on day 07 were 2.2, 2, and 6.5%, respectively. These rates gradually increased to 21.1, 25, and 24.1%, respectively, on day 28. This suggests that conjugation with trypsin and pepsin did not alter the degradation rate of PNF. Amongst all, Pan-PNF showed the highest degradation potential of 34.6% on day 28. These findings suggest that PNF is a polymer with slow degradation potential that may be employed in long-term therapeutic applications.

### 3.3. Mechanical Characterization

**Figure 8:**
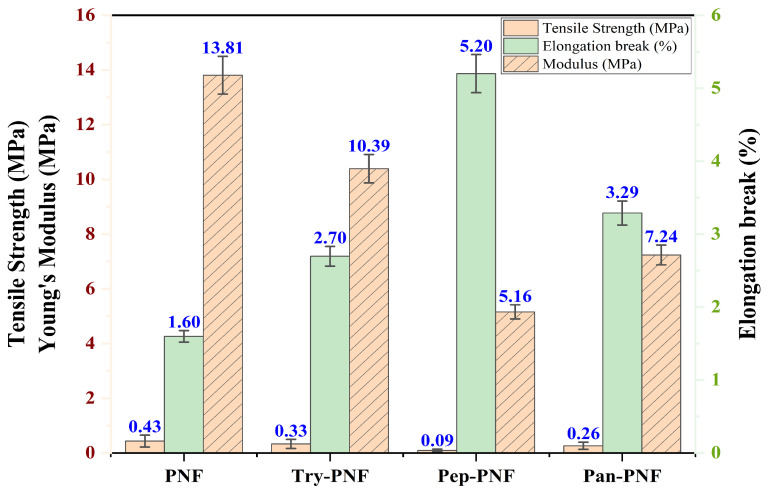
Tensile strength and elongation break (%) of PNF, Try-PNF, Pep-PNF, and Pan-PNF.

Mechanical characteristic evaluation of PNF, Try-PNF, Pan-PNF, and Pep-PNF, as represented in Figure 8, suggests that PNF displayed the most elevated tensile strength of 0.43 ± 0.16 MPa, indicating it is most capable of withstanding applied stress without failure. Try-PNF and Pan-PNF revealed moderate tensile strength of 0.33 ± 0.21 MPa and 0.264 ± 0.13 MPa, whereas Pep-PNF had a lower tensile strength of 0.092 ± 0.046 MPa. Likewise, with Young’s modulus, PNF again had the highest stiffness of 13.81 ± 0.69 MPa, indicating a rigid scaffold structure, and Pep-PNF had the low rigidness of 5.16 ± 0.51 MPa. PNF scaffold flexibility was estimated in terms of elongation at break, with PNF having 1.60 ± 0.13%, followed by Try-PNF, Pep-PNF, and Pan-PNF, depicting narrow stretching and indicating poor flexibility. These results reveal that PNF (*p* < 0.05) acts as a promising candidate with notable strength, flexibility, and rigidity. In contrast, enzyme-conjugated scaffolds may require further optimization for clinical applications. These results furnish a mechanical basis for additional biological evaluation and nanofiber scaffold design techniques.

### 3.4. Biochemical Characterization of PNFs

#### 3.4.1. Protease Activity Assay

**Figure 9:**
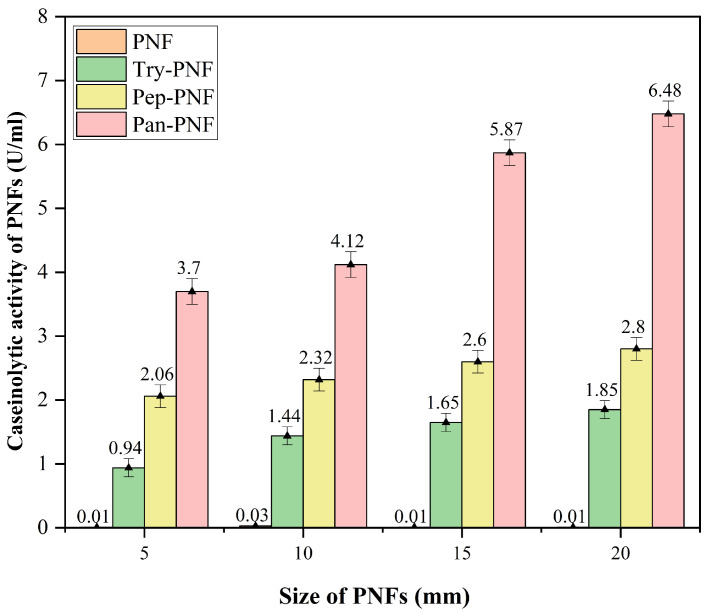
Protease activity of the nanofibers, Pan-PNF showing the highest proteolytic activity among all other PFs.

Enzyme-conjugated polycaprolactone nanofiber discs of 5 mm, 10 mm, 15 mm, and 20 mm were investigated for size-dependent proteolytic efficiency. As shown in Figure 9, plain PNF did not show any proteolytic activity, while trypsin-, pancreatin-, and pepsin-conjugated PNFs displayed increased proteolytic activity in a size-dependent manner. Enzymatic activity of Try-PNF, Pan-PNF, and Pep-PNF ranged between 0.94 ± 0.01 and 1.85 ± 0.52 U, 3.7 ± 0.17 U and 6.48 ± 0.20 U, and 2.06 ± 0.34 U and 2.8 ± 0.19 U, respectively. Increasing the size (5–20 mm) of discs showed no significant boost in activity. In addition, Pan-PNF and Try-PNF activity increased 2-fold between 5 mm and 20 mm scaffolds, while Pan-PNF (*p* < 0.05) alone showed 3–5-fold increased activity compared to 20 mm Pep-PNF and Try-PNF discs.

#### 3.4.2. Effect of pH on the Activity of the Immobilized Enzyme

**Figure 10:**
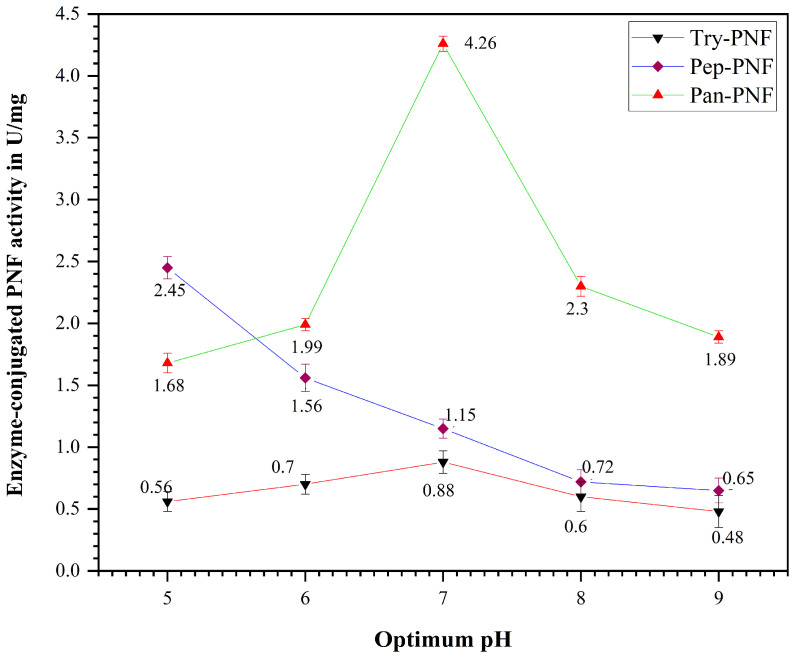
Optimum pH of the enzyme-conjugated PNF (Try-PNF, Pep-PNF, and Pan-PNF).

Enzyme-conjugated polycaprolactone nanofibers were incubated using various pH buffers, and their proteolytic efficiency was investigated at different pH ranges (5, 6, 7, 8, and 9). The enzyme activity increases with rising pH values until they reach a maximum (optimum pH), after which further increase in pH results in decreased activity. A similar trend (Figure 10) was observed in Try-PNF and Pan-PNFs (0.88 ± 0.18 U and 4.26 ± 0.10 U) activity at an optimum pH of 7.0. It was evident that activity was decreased at pH 5, 6, 8, and 9. This indicates that these enzymes are active in neutral environments. Nonetheless, Pep-PNFs displayed notable proteolytic activity over wider pH ranges from 5, 6, and 7 (2.45 ± 0.10 U, 1.99 ± 0.15 U, and 1.15 ± 0.06 U) and were less active at alkaline conditions.

#### 3.4.3. Effect of Temperature on the Activity of the Immobilized Enzyme

**Figure 11:**
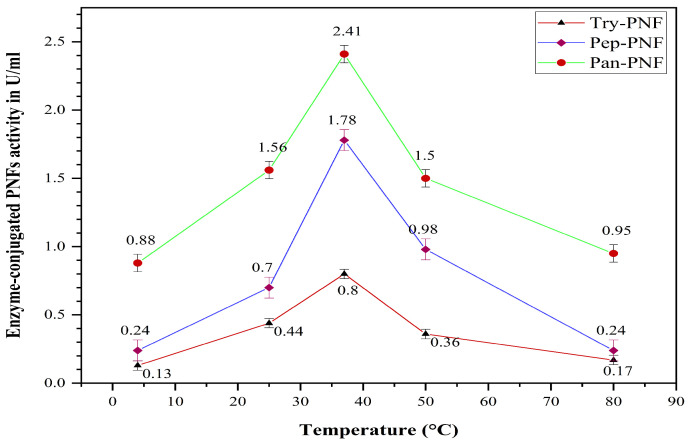
Optimum temperature of the enzyme-conjugated PNF (Try-PNF, Pep-PNF, and Pan-PNF).

Try-PNF, Pan-PNF, and Pep-PNF were evaluated for their thermal stability and enzyme activity over a wide range of temperatures: 4 °C, 25 °C, 37 °C, 50 °C, and 80 °C. At 37 °C, Pan-PNF displayed high proteolytic activity of 2.41 ± 0.26 U and, at 4 °C, the lowest enzyme activity of 0.88 ± 0.45 U was observed (Figure 11). Furthermore, at 50 °C and 80 °C, notable proteolytic activity of 1.50 ± 0.21 U and 0.95 ± 0.18 U was observed, respectively, indicating good thermal stability. Proteolytic trends of Try-PNF were similar to that of Pep-PNF, with peak activity at 37 °C (0.8 ± 0.37 U and 1.78 ± 0.70 U, respectively), followed by a decline in proteolysis with rising temperature.

## 4. Discussion

Polycaprolactone nanofibrous membrane conjugation with enzymes (trypsin, pepsin, and pancreatin) significantly improves various physicochemical properties such as porosity, wettability, rate of degradation, thermal and ionic stability, and morphological features of nanofibers for biomedical applications. In the current study, it was evident that PNFs conjugated with enzymes have proven to increase the catalytic efficiency of PNF itself. Current results are consistent with results from a previous study [32], proving active enzyme conjugation. Additionally, EDX elemental analysis and FTIR functional group data indicate successful conjugation of enzymes [33].

Surface modifications of nanofibers result in increased hydrophilicity, making them more compatible for biomedical applications. Excessive hydrophobicity of nanofibrous membranes can hinder cell adhesion, proliferation, and nutrient exchange, critical factors in wound healing and tissue regeneration [34,35]. As remarked by Samadian et al. (2020) [36], elevated wettability and porosity of surface-modified nanofibrous membranes are consistent to results observed for Pep-PNFs, making them excellent candidates for controlled drug delivery, wet wounds, absorption of exudates, and areas requiring cell–cell interactions. In particular, the improved hydrophilicity observed post-conjugation suggests a more favorable environment for cellular interactions, which aligns with the intended application in tissue engineering and wound healing platforms [36]. In contrast, Pan-PNF can be employed in areas where limited absorption and slower drug release are preferred [34]. Although the current findings provide a general insight into the solid–liquid interactions between the nanofibers and liquid components, comprehensive surface energy analyses using various test liquids are underway to more precisely elucidate the wettability characteristics and interfacial behavior of the nanofiber surfaces.

Furthermore, the lower tensile strength of PNF scaffolds (0.09–0.43 MPa) may be attributed to factors such as a highly porous structure and irregularly aligned fibers, the conditions that may vary from physiological conditions. Despite these drawbacks, low-tensile-strength PCL scaffolds still hold promising applications in enzyme immobilization or drug delivery systems, as reported earlier [37]. However, further optimization is necessary to increase tensile strength, together with successful conjugation with enzymes. 

The degradation study shows that the potential of Try-PNF and Pep-PNF is marginally higher than PNF alone. Pan-PNF, of all PNFs, is formed by the combination of multiple digestive enzymes, thus resulting in a relatively higher rate of degradation of polycaprolactone nanofibers. These findings are consistent with the previous report by Khan et al. (2019) [38] findings, which noted that polycaprolactone nanofibers are formed by various bonds such as ester bonds, disulfide bonds, hydrogen bonds, and other non-covalent bonds, which are highly specific sites for enzyme-mediated degradation. Fast degradation of Pan-PNF and slow degradation of PNF may serve as vital candidates for diverse applications requiring rapid biodegradable materials and long-term care endurable materials, respectively.

Likewise, Pan-PNF (multi-catalytic enzymes) proteolytic activity was higher compared to Try-PNF and Pep-PNF. Lower activity of Pep-PNF may be affected by varying hydrogen ion concentration, while trypsin and pancreatin activity was influenced by the availability of active sites and substrates, larger surface area, and spatial arrangement of enzymes onto the scaffolds [39,40,41]. The proteolytic activity of Pan-PNF and Pep-PNF was relatively higher, which is consistent with earlier studies, in which pepsin and pancreatin are emphasized as notable proteases for protein degradation [42,43]. However, apart from qualitative analysis, quantitative yield/efficiency of enzymes alone versus enzyme-conjugated scaffolds would have provided additional evidence in describing the influence of scaffolds on enzyme activity. Further study will be focused on the yield ratio of enzymes, the use of different substrates, and analyzing degradation products with varying physical factors to investigate their efficiency in wound environments. These biocompatible PNFs with protein degradation potential may be of great importance in biomedical applications such as targeted enzyme therapy, drug delivery, and wound dressings.

The activities of enzymes such as trypsin, pancreatin, and pepsin were consistent with their respective physiological optimum pH values. Trypsin is known to be fully functional around neutral pH values, like pancreatin. Severe decline in enzyme activity is caused by compromised proteins’ functional and structural integrity [44]. The activity of pepsin PNFs confirms their catalytic efficiency in acidic environments (pH 5). This wide array of enzyme-conjugated PNFs can be fabricated and tuned as per therapeutic needs to avail them at their maximum potential. These findings suggest that Pep-PNFs active at an acidic pH range can be employed to treat various kinds of wounds constituting necrotic tissues, diabetic ulcers, and sores, as these wounds usually have acidic and fluctuating pH environments [45]. Thus, Pep-PNFs can be used as wound debridement agents to facilitate a healthy healing process and minimize risks. Try-PNF and Pan-PNF can be employed in neutral to alkaline wound environments. However, controlled management of PNFs is crucial in maintaining intact activity at chronic wound sites.

Temperature also plays a crucial role in enzyme activity. Try-PNF demonstrated a substantial reduction in activity with increasing temperatures, implying it is thermolabile compared to Pep-PNF, while Pan-PNF is thermally stable. These findings reveal that enzymes are thermally sensitive, and the enzyme activity is dependent on temperatures. The declining activity of enzymes as the temperature rises may be due to the denaturation of protein structures and loss of integrity. The Pan-PNF displayed strong thermal stability, which may be attributed to the fact that conjugation with polymers provides enzymes with more structural and functional stability [46]. These thermostable enzyme–polymer conjugates play a crucial role in industrial applications, chronic necrotic wounds, and diagnostic therapeutics [47].

Overall, each factor determines the efficiency of enzymes; thus, selective surface modification of these nanofibrous membranes yields enhanced results, increasing their overall potential for tissue engineering and other biomedical applications. Current study focuses on the fabrication and characterization of nanofiber scaffolds conjugated with various digestive enzymes, but various characterization studies, such as X-ray photoelectron spectroscopy, attenuated total reflectance–Fourier transform infrared spectroscopy, and antimicrobial and cell culture studies, are in progress to evaluate the potential of scaffolds for practical applications.

## 5. Conclusions

In this study, a novel way of fabricating enzyme-incorporated polycaprolactone nanofibers for tissue engineering scaffolds, enzyme therapy, or drug delivery was explored. These enzyme-incorporated PNFs (Try-PNF, Pep-PNF, and Pan-PNF) have not been previously explored in this context. This study also emphasizes a critical research gap by addressing therapeutic potential of readily available proteolytic enzymes in nanofiber scaffolds over the commonly explored lipase-based polymeric systems. Characterization of enzyme-conjugated PNF indicated unique chemical and physical property transformations compared to PNF alone. The nanofibers’ strength, chemical composition, hydrophilicity, porosity, and degradation rates were highly influenced by enzyme conjugates. Such modified PNFs possess vast applications in the treatment of moist wounds with higher rates of wound exudates. These tunable PNFs are promising therapeutic substitutes as drug delivery agents in wound healing, targeted therapy, and tissue engineering and infection control. Future studies must focus on the optimization of these PNFs in in vitro and in vivo studies to assess clinical efficiency. This study lays a foundation for the development of innovative wound dressings combining mechanical aspects of nanofibers with biochemical aspects of enzymes, thus demonstrating promising therapeutic options for a wide array of wounds and wound conditions.

## Figures and Tables

**Table 1:** EDX elemental analysis of PNFs (uncertainty analysis report ≈ 10.6).

PNFs	Element	Weight %	Atomic %	Error %
PNF	C K	66.8	72.8	9.0
	O K	33.2	27.2	11.8
Try-PNF	C K	58.9	65.3	9.2
	N K	4.4	4.2	21.1
	O K	36.7	30.5	11.6
Pep-PNF	C K	61.2	67.3	9.1
	N K	5.1	4.8	20.1
	O K	33.8	27.9	11.8
Pan-PNF	C K	59.5	65.8	9.1
	N K	5.0	4.8	21.7
	O K	35.5	29.4	11.7

**Table 2:** Contact angle measurements of nanofibrous scaffolds.

Nanofibrous Scaffolds	Left Angle (Θ)	Right Angle (Θ)
PNF	87.8 ± 2.3	92.2 ± 1.3
Try-PNF	88.0 ± 2.1	88.7 ± 1.8
Pep-PNF	57.6 ± 2.3	57.9 ± 2.5
Pan-PNF	67.2 ± 2.2	65.9 ± 2.6

## Data Availability

Data available on request from the corresponding author as the data is part of ongoing study.

## Abbreviations

The following abbreviations are used in this manuscript:

CBB	Coomassie Brilliant Blue
EDX	Energy-dispersive X-ray
EtOH	Ethyl alcohol
FE-SEM	Field Emission Scanning Electron Microscope
FTIR	Fourier Transform Infrared Spectroscopy
Pan-PNF	Pancreatin-conjugated Polycaprolactone Nanofiber
PCL	Polycaprolactone
Pep-PNF	Pepsin-conjugated Polycaprolactone Nanofiber
PNF	Polycaprolactone Nanofiber
SEM	Scanning Electron Microscope
TCA	Trichloroacetic acid
Tris–HCl	Tris(hydroxymethyl)aminomethane hydrochloride
Try-PNF	Trypsin-conjugated Polycaprolactone Nanofiber
