# Correction: Shivakumar et al. Synthesis, Characterization, and Enzyme Conjugation of Polycaprolactone Nanofibers for Tissue Engineering. *Pharmaceutics* 2025, *17*, 953

**DOI:** 10.3390/pharmaceutics18020234

**Published:** 2026-02-12

**Authors:** Chandana B. Shivakumar, Nithya Rani Raju, Pruthvi G. Ramu, Prashant M. Vishwanath, Ekaterina Silina, Victor Stupin, Raghu Ram Achar

**Affiliations:** 1 Division of Biochemistry, School of Life Sciences, Mysuru, JSS Academy of Higher Education and Research, Mysuru 570015, Karnataka, India; chandanabs@jssuni.edu.in (C.B.S.);; 2 Department of Biochemistry, JSS Medical College, JSS Academy of Higher Education and Research, Mysuru 570015, Karnataka, India; 3 I.M. Sechenov First Moscow State Medical University (Sechenov University), 119991 Moscow, Russia; 4 Department of Hospital Surgery, Pirogov Russian National Research Medical University, 117997 Moscow, Russia; 5 Department of Biochemistry, Mahayogi Gorakhnath University Gorakhpur, Gorakhpur 273007, Uttar Pradesh, India

## Error in Figure

In the original published paper [1], there was a mistake in “Figure 4” as published. The panel labeled (iv), which was intended to represent the Pan-PNF sample, was inadvertently substituted with the image of Figure 4ii Try-PNF during figure preparation and layout. The corrected Figure 4 appears below. The authors state that their scientific conclusions are unaffected. This correction was approved by the Academic Editor. The original publication has also been updated.

## Figures and Tables

**Figure 4:**
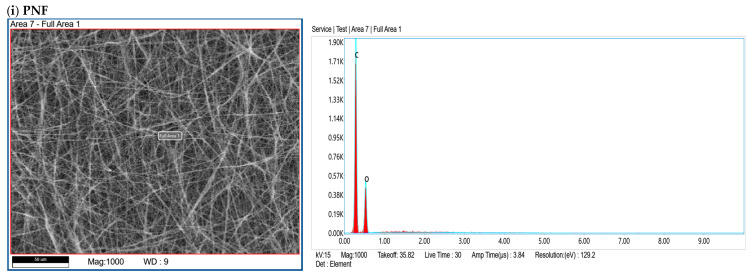

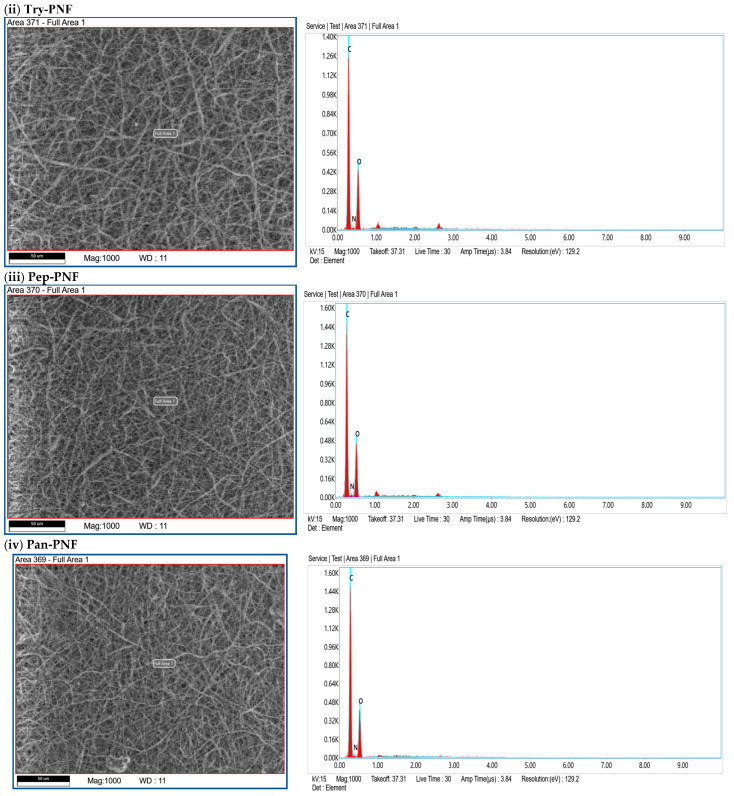
EDX elemental analysis of PNFs showing the confirmation of the enzyme’s conjugation by the presence of nitrogen and increased level of oxygen.
